# Cross-Species Gene Expression Analysis Reveals Gene Modules Implicated in Human Osteosarcoma

**DOI:** 10.3389/fgene.2019.00697

**Published:** 2019-08-07

**Authors:** Zheng Jin, Shanshan Liu, Pei Zhu, Mengyan Tang, Yuanxin Wang, Yuan Tian, Dong Li, Xun Zhu, Dongmei Yan, Zhenhua Zhu

**Affiliations:** ^1^Department of Immunology, College of Basic Medical Sciences, Jilin University, Changchun, China; ^2^Key Laboratory for Molecular Enzymology and Engineering, Ministry of Education, Jilin University, Changchun, China; ^3^Department of Orthopaedic Trauma, The First Hospital of Jilin University, Changchun, China

**Keywords:** osteosarcoma, hub genes, cross-species analysis, WGCNA, CD86

## Abstract

**Background:** Osteosarcoma (OS) is one of the malignant bone tumors occurring in both human and canine, and in both of them, it is characterized by a high rate of metastasis and poor prognosis. Cross-species analysis reveals previously neglected molecular or signaling pathways involved in the progression of diseases, and dogs are genetically comparable to humans and live in similar environments. Therefore, the aim of this study was to find out OS hub genes through a cross-species analysis.

**Materials and Methods:** All the human and canine OS gene expression data obtained by the Affymetrix platform were collected. After quality assessment and normalization, co-expression network was performed using weighted gene co-expression network analysis (WGCNA). Species-specific modules and consensus modules were identified. Protein–protein interaction (PPI) networks analysis was performed based on consensus gene modules. Then, consensus modules were functionally annotated and correlated with clinical traits. Hub nodes were identified by a subnetwork analysis of PPI network and WGCNA module membership. Modules of interest and hub nodes were validated in an external data set.

**Results:** Three modules for the human network, seven modules for the canine network, and four consensus modules were identified. The consensus module 3 (C3) showed a significant correlation with the metastatic status in the training data set and a significant correlation with metastasis-free survival in the external data set. Cluster of differentiation 86 (CD86) was identified as the hub gene of C3, showing a significant correlation with metastasis-free survival.

**Conclusion:** Genes in C3 play an important role in OS metastasis, whereas CD86 might be a potential molecular biomarker for OS metastasis.

## Introduction

Osteosarcoma (OS) is one of the most common malignant bone tumors in children and adolescents (Broadhead et al., 2011), arising from primitive mesenchymal bone-forming cells that exhibit osteoblastic differentiation (Luetke et al., 2014). The incidence of OS is one to three cases per million worldwide (Kerr et al., 2013). OS is a highly invasive cancer, and it is characterized by an early systemic metastasis (Messerschmitt et al., 2009). Approximately 20% of patients have metastases at first diagnosis, and approximately 50% have lung metastases at the late stage of the disease (Daw et al., 2015). Over the past 30 years, the 5-year survival rate has increased from 10% to 70% for non-metastatic patients thanks to the combination treatment of surgery and chemotherapy (Longhi et al., 2006). However, the prognosis of patients with metastatic OS remains very poor. Even after this combined treatment, only 11% to 30% of patients with OS metastases survive (Chou et al., 2005). Therefore, it is necessary to improve the understanding of the biological process and metastasis of OS to enable a correct OS diagnosis as early as possible and improve its treatment.

Canines are characterized by naturally occurring tumors, providing an opportunity to study human tumors since dogs are genetically comparable to humans and live in similar environments. OS is one of the most common malignant tumors in dogs, and shows similarities with human OS regarding gene features, tumor radiological features, clinical features, and metastatic patterns (Shao et al., 2018).

Cross-species research reduces the impact of individual differences on conclusions and increases the understanding of a disease. Cross-species analysis yielded good results in glioma (Miller et al., 2010), osteoarthritis (Mueller et al., 2017), and insulin resistance (Chaudhuri et al., 2015), revealing previously neglected molecular or signaling pathways involved in the progression of these diseases. At present, several studies focused in the analysis of OS across species. Paoloni et al. (2009) sequenced 15 canine OS samples and 15 human OS specimens to obtain metastasis-associated tumor targets. Humans and dogs are highly similar regarding gene expression and clustering. For example, both IL-8 and SLC1A3 are biomarkers of poor prognosis in both dogs and humans. Allen-Rhoades et al. (2015) used 14 mouse models of OS, serum was extracted, and miRNA sequencing was performed to discover prognostic miRNAs that were actually verified in 40 human patients with OS, additionally revealing that miRNA-204 is a good prognostic molecule. In Shao et al. (2018) study, 52 human OS specimens and 9 dog OS specimens were analyzed, and the results revealed that the copy number of DLG2 in both dogs and humans was significantly reduced, suggesting that DLG2 may be a potential target for the inhibition of OS.

Weighted gene co-expression network analysis (WGCNA) (Zhang and Horvath, 2005) is a system biology method that takes into account the correlation between genes. The core idea of WGCNA is that it is not a single gene, but a group of genes with similar expression patterns under specific circumstances that exert certain biological effects. This idea was tested in a variety of biological processes (Langfelder and Horvath, 2008). Specifically, a co-expression network was constructed among all genes, and genes with similar expression patterns were classified as belonging to the same module. Then, the correlation between the module and clinical features was analyzed, resulting in the discovery of modules highly related to clinical traits. Due to the connectivity of the genes within the module, they were analyzed to discover genes with clinical significance. Therefore, this method allows the identification of genes that are biologically relevant and yields meaningful results in multiple cross-species studies (Miller et al., 2010; Mueller et al., 2017).

Thus, the aim of this study was to collect the maximum number of available mRNA expressing data related to samples from humans and dogs. WGCNA was used to analyze and discover the conservative gene expression modules between human and canine. The correlation between the module and clinical trait was evaluated to assess the function of the genes within the module. Therefore, the knowledge of the OS transcriptome became more profound, thus providing new approaches in the clinical treatment of this disease.

## Materials and Methods

### Data Collection, Merging, and Standardization

An overview of the data processing is shown in **Supplementary Table 1**. Gene Expression Omnibus (http://www.ncbi.nlm.nih.gov/geo/) and ArrayExpress (http://www.ebi.ac.uk/arrayexpress/) were used to obtain OS data, and the maximum number of available Affymetrix platform data referred to human and dog specimens or cell lines were collected. The inclusion criteria were the following: 1) The data set should contain OS specimens or cell lines, and at least three biological replicates; 2) OS cell lines were not subjected to any treatment with drugs or other factors; 3) raw cell data should be available. All raw data were processed using R software. The background of each data set was corrected using the RMA algorithm (Irizarry et al., 2003). Then, the expression of each data set was normalized using the function “normalizeCyclicLoess” of the “limma” package in R. The probes were annotated according to entrez ID on the basis of the specific platform used by the data set. If multiple probes corresponded to the same entrez ID, the one with the highest average expression value was selected by the “collapseRows” function of WGCNA (Miller et al., 2011). Expression matrices were merged according to the common entrez ID in human data sets or canine data sets separately. As regard human data sets, entrez IDs were reannotated to Ensembl gene ID using biomaRt (Durinck et al., 2005) package. As regard canine data sets, entrez IDs were reannotated to human Ensembl gene ID that has a homologous sequence with canine using biomaRt (Durinck et al., 2005) package. The batch effect among different studies was removed using the SVA software package (Chakraborty et al., 2012) in human and canine data sets separately, and then, the normalization of the two merged expression matrices was separately performed using Z-score in the “data.Normalization” function in R package CancerSubtypes, to obtain comparable data (Xu et al., 2017).

### WGCNA

Genes were selected according to the common ID in the two merging matrices, and the ones that passed the quality test in human and dog species by the “goodGene” function in WGCNA were selected for further analysis. The connectivity of all samples was separately calculated in the human merged matrix and canine merged matrix. Samples with a connectivity z.k < −2.5 were screened out as outliers. The minimum soft threshold that formed a scale-free network in both human and canine matrix was screened out using the “pickSoftThreshold” function in the WGCNA package. Next, the adjacency matrix was separately calculated in the two merged matrices and dissimilarity was calculated as 1-adjacency. To identify co-expression modules, genes were clustered according to the dissimilarity, and gene modules were identified using a dynamic tree cut method developed by Langfelder (Horvath and Dong, 2008). Co-expression gene modules for human and canine were separately calculated under the same parameters. Consensus dissimilarity of human and canine merged gene expression data was used to define consensus gene modules. In comparison to single species gene modules, consensus modules obtained in this work contained genes that were closely related to both species. In human, canine, or consensus network, modules were identified with colors at a specific order in accordance with the module size at the specific network. Preservation test was conducted to evaluate whether modules were preserved across species.

### Module–Trait Relationship

Module eigengene (ME) is defined as the first principal component of a gene module (Zhang and Horvath, 2005). Spearman correlation between each consensus MEs and clinical traits was calculated to find modules of interest.

### Network Visualization and Annotation

Relationship among genes in the consensus modules was visualized using Cytoscape (Cline et al., 2007). Genes in the module were imported into the String V10.5 (https://string-db.org) (von Mering et al., 2003) to build the protein interaction network according to the protein–protein interaction (PPI) relationship among module genes. Next, “clusterProfiler” package in R (Yu et al., 2012) was used to annotate and visualize gene function in modules. To screen the hub genes, the module network and PPI network were first imported into Cytoscape (Shannon et al., 2003), a network visualization platform. Then, using MCODE (Bader and Hogue, 2003) plug-in of Cytoscape, subnetworks of both module and PPI network were identified at default parameters. Each subnetwork was scored according to the connectivity of the whole subnetwork. The subnetwork with the highest score was defined as the hub network. Nodes that appeared in both the module hub network and PPI hub network were defined as hub genes.

### Survival Analysis

The external data set GSE21257 (Buddingh et al., 2011), which was generated on Illumina platform, contained 53 OS samples and associated metastasis information, and was used to evaluate the significance of consensus MEs in the metastasis. Samples were divided into two groups, high and low, based on the expression of consensus MEs, in comparison to the median ME level to test the survival significance of consensus MEs. Then, hub genes survival significance was evaluated in both training data set and testing data set.

#### Network Validation in Mouse Species

To test whether the consensus network identified between human and canine was stable in multiple species, preservation analysis of human-canine consensus modules in the mouse expression data was conducted, then similar processes were performed to construct the human-mouse consensus network. The external data set GSE87685 (Scott et al., 2018), which was generated on Illumina platform, contained 103 mouse OS samples and was used to construct the consensus network. After the mouse OS expression data were Z-score normalized, common genes between human and mouse samples were selected. Preservation test was used to test whether the relationship of genes in human–canine consensus modules reappeared in mouse data set. Under the soft power of 7, same to the human–canine consensus network, the human–mouse consensus network was constructed. The co-expression networks of human–canine and human–mouse were compared, and the survival significance of the overlapped modules was verified in the external data set GSE21257.

## Results

### Construction of Canine and Human Co-Expression Network

**Figure 1:**
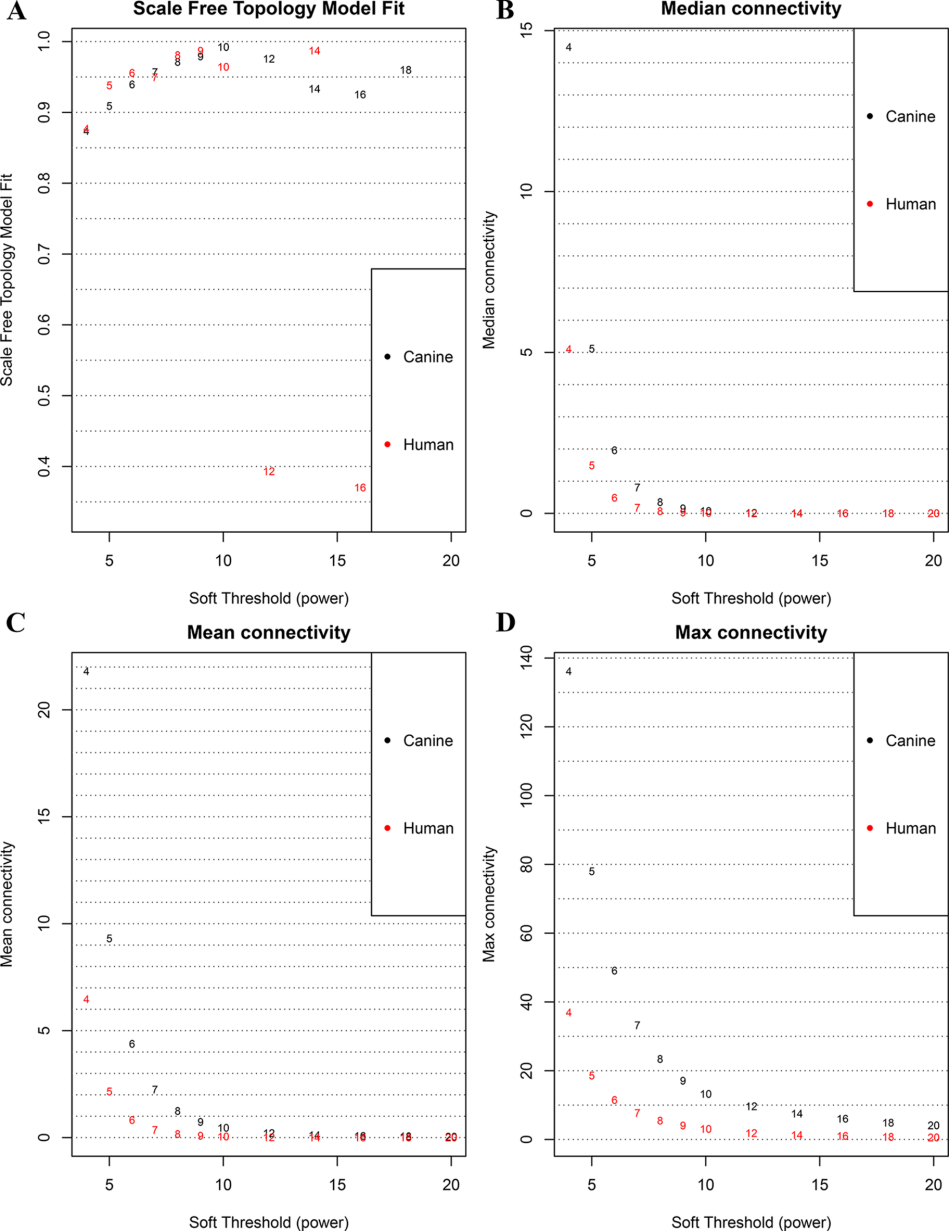
Correlation between soft power and network connectivity. **(A)** human and canine network could reach a scale-free network (y-axis, R^2^ > 0.95) when a soft threshold was set to 7. **(B**, **C**, **D)** median connectivity and mean connectivity revealing that both human and canine network showed negligible connectivity, max connectivity revealing that only a small amount of nodes showed a relative high connectivity when the soft threshold was set to 7. This figure shows that when the soft threshold is 7, both networks present the characteristic of scale-free network distribution.

**Figure 2:**
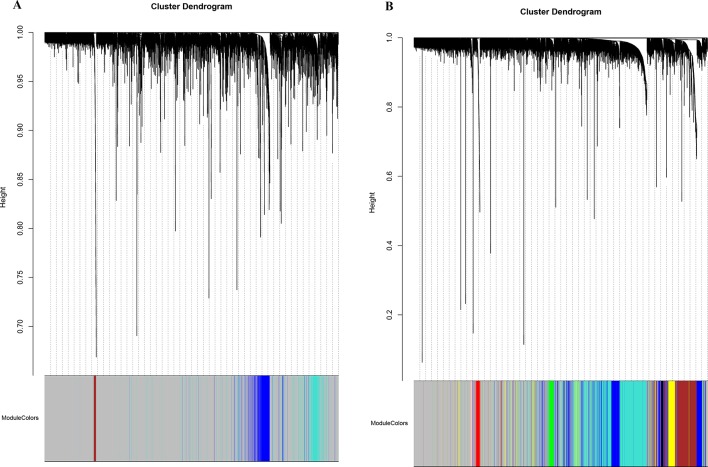
Network cluster dendrogram. Human (three co-expression modules [H1-H3]) **(A)** and canine (eight co-expression modules [CA1-CA8]) **(B)**, in which a color was assigned to each module. The color grey was assigned to genes that could not cluster into a specific module.

The database retrieval resulted in 154 human samples (18 cell lines and 136 tumor specimens) in nine data sets (Paoloni et al., 2009; Sadikovic et al., 2009; Fritsche-Guenther et al., 2010; Kobayashi et al., 2010; Vella et al., 2016) and 117 canine samples (11 cell lines and 106 tumor specimens) in five data sets (Paoloni et al., 2009; Scott et al., 2011; Fowles et al., 2016), and all of them were initially included in this study (**Table 1**). The expression data were annotated, merged, and standardized, and WGCNA screened out eight human samples and three canine samples as outliers. As a result, 146 human samples and 114 canine samples with 4,609 common genes were considered and subjected to WGCNA analysis. Expression profiles of human and canine species were visualized in a PCA plot (**Supplementary Figure 1**), and no clustering was found in the samples. A soft power of seven was selected to construct a scale-free network (**Figure 1**). Finally, three co-expression modules (H1–H3) were constructed through the human samples considered, and eight co-expression modules (CA1–CA8) were constructed through the canine samples considered (**Figure 2**). Cross-species preservation analysis was conducted to test whether human or canine co-expression modules were preserved across the two species. All the three human co-expression modules showed a considerable preservation (Preservation Zsummary > 10) in canine. The canine modules, except for the green and pink modules, showed a high preservation (Preservation Zsummary > 10) in human (**Supplementary Figure 2**). Genes assigned to each human or canine module are provided in the **Supplementary Sheets 1–11**.

**Table 1:** Information of datasets used in this study.

ID	Species	Platform	Cell line	Samples	Primary/Metastasis
GSE12865 ^28^	Human	HuGene-1_0-st	0	12	12/0
GSE14359 ^29^	Human	HG-U133A	0	18	10/8
GSE14827 ^30^	Human	HG-U133_Plus_2	0	27	27/0
GSE16088 ^12^	Human	HG-U133A	3	14	14/0
GSE16091 ^12^	Human	HG-U133A	0	34	34/0
GSE39262	Human	HG-U133A	10	0	0/0
GSE70414	Human	HG-U133_Plus_2	5	0	0/0
GSE73166	Human	HuGene-1_0-st	0	10	3/7
GSE87437 ^31^	Human	HG-U133_Plus_2	0	21	21/0
GE16087 ^12^	Canine	Canine	2	15	NA
GSE27217 ^32^	Canine	Canine_2	28	6	NA
GSE57884	Canine	Canine_2	6	0	0/0
GSE63476	Canine	Canine_2	0	18	NA
GSE76128 ^33^	Canine	Canine_2	9	33	NA

There were five datasets without associated citation information in GEO database.

### Consensus Modules Established Between Species

**Figure 3:**
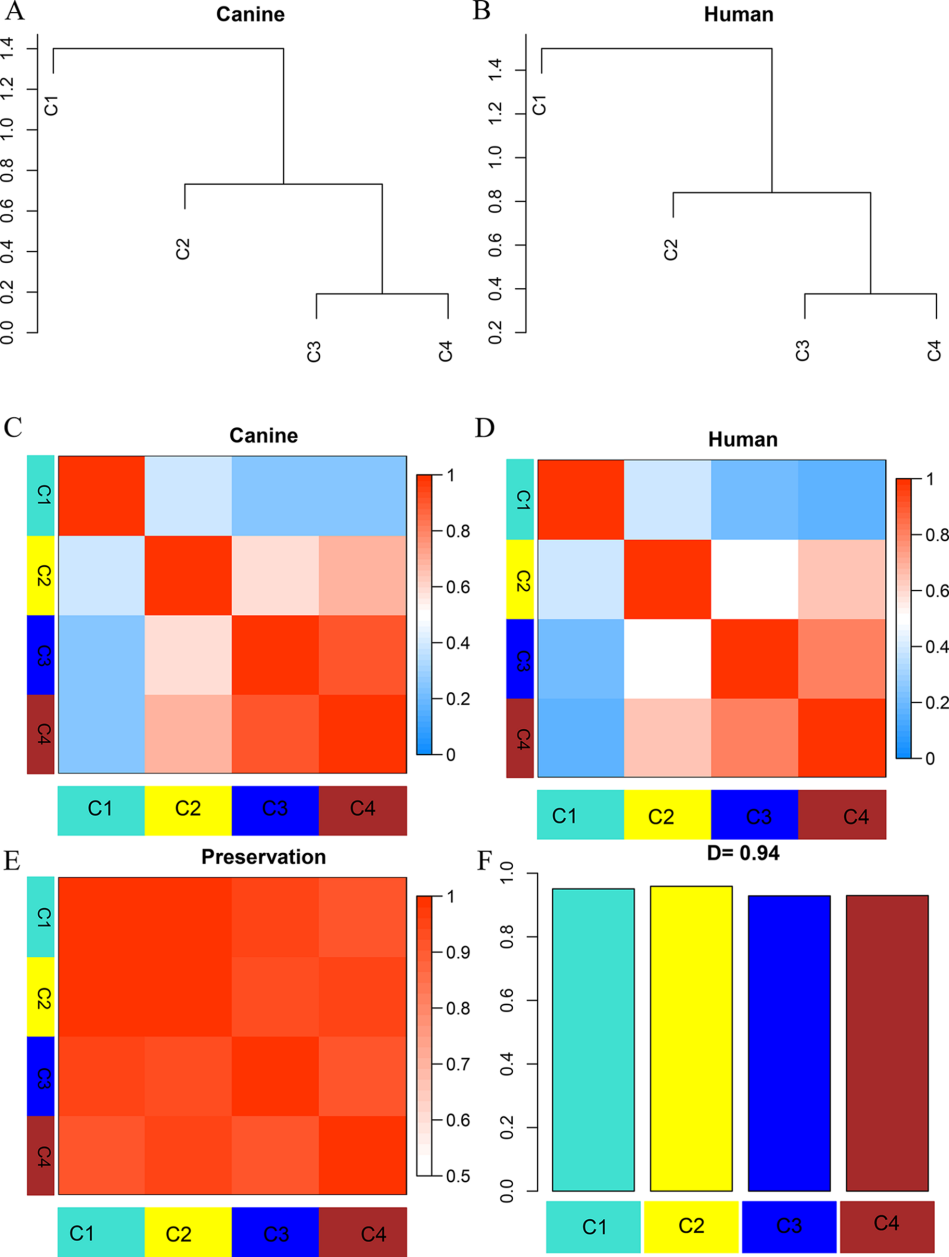
Dendrogram and eigengene representation of consensus eigengene network. **(A**, **B)** The same branches were found in both species’ dendrograms. **(C**, **D)** Heatmap of eigengene adjacencies for each species **(C**: canine; **D**: human), the red color indicates high adjacency (positive correlation), the blue indicates low adjacency. **(E)** Adjacency heatmap for the pairwise preservation between the two networks, the red color indicating a high preservation between the two networks. **(F)** Barplot of the preservation between the two networks. The high density value D = 0.94 reflects a high overall preservation between the two networks.

**Figure 4:**
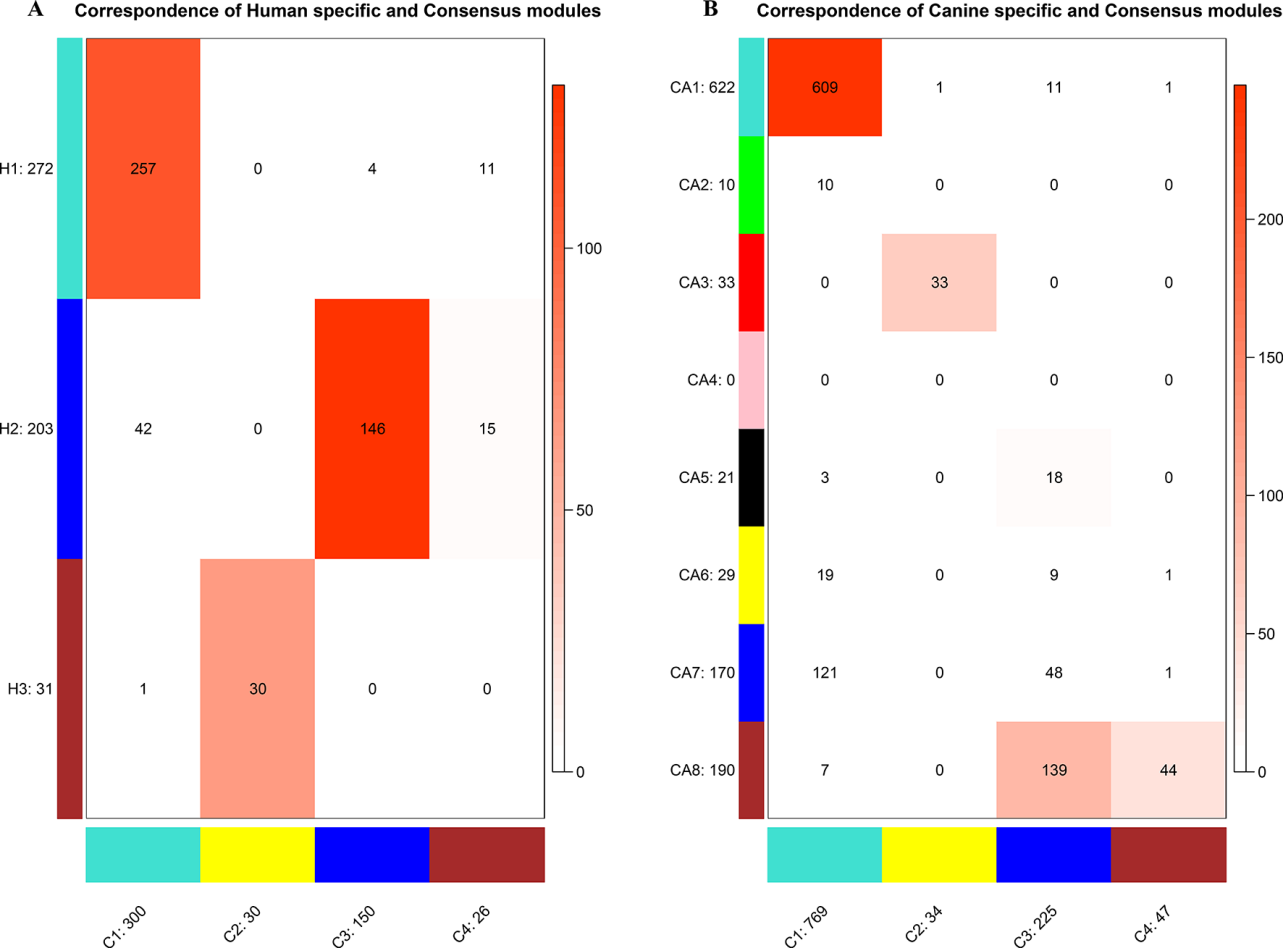
Correspondence of human **(A)** or canine **(B)** modules and consensus modules. The colored cell indicates a significant overlap and the darker the color, the higher the gene overlap. H1 showed a significant overlap with C1. H2 showed a significant overlap with C3 and C4. H3 showed a significant overlap with C2. CA1 showed a significant overlap with C1. CA3 showed a significant overlap with C2. CA5 showed a significant overlap with C3. CA8 showed a significant overlap with C3 and C4.

To establish the consensus modules between the two species that were considered, such as the modules shared by the two species (canine and human), the weight average correlation matrix was adopted. Four consensus modules (C1–C4) came out as a result. These consensus modules showed a high preservation across species and a similar cluster structure (**Figure 3**). Genes assigned to each consensus modules are provided in the **Supplementary Sheets 12–15**. Human modules H1, H2, H3 showed a significant overlap with C1, C3, C2, respectively (**Figure 4A**). Canine modules, CA1, CA3 showed a significant overlap with C1, C2, respectively. CA8 showed a significant overlap with both C3 and C4 modules (**Figure 4B**).

### Module–Trait Relationships Defined Clinical-Associated Modules

**Figure 5:**
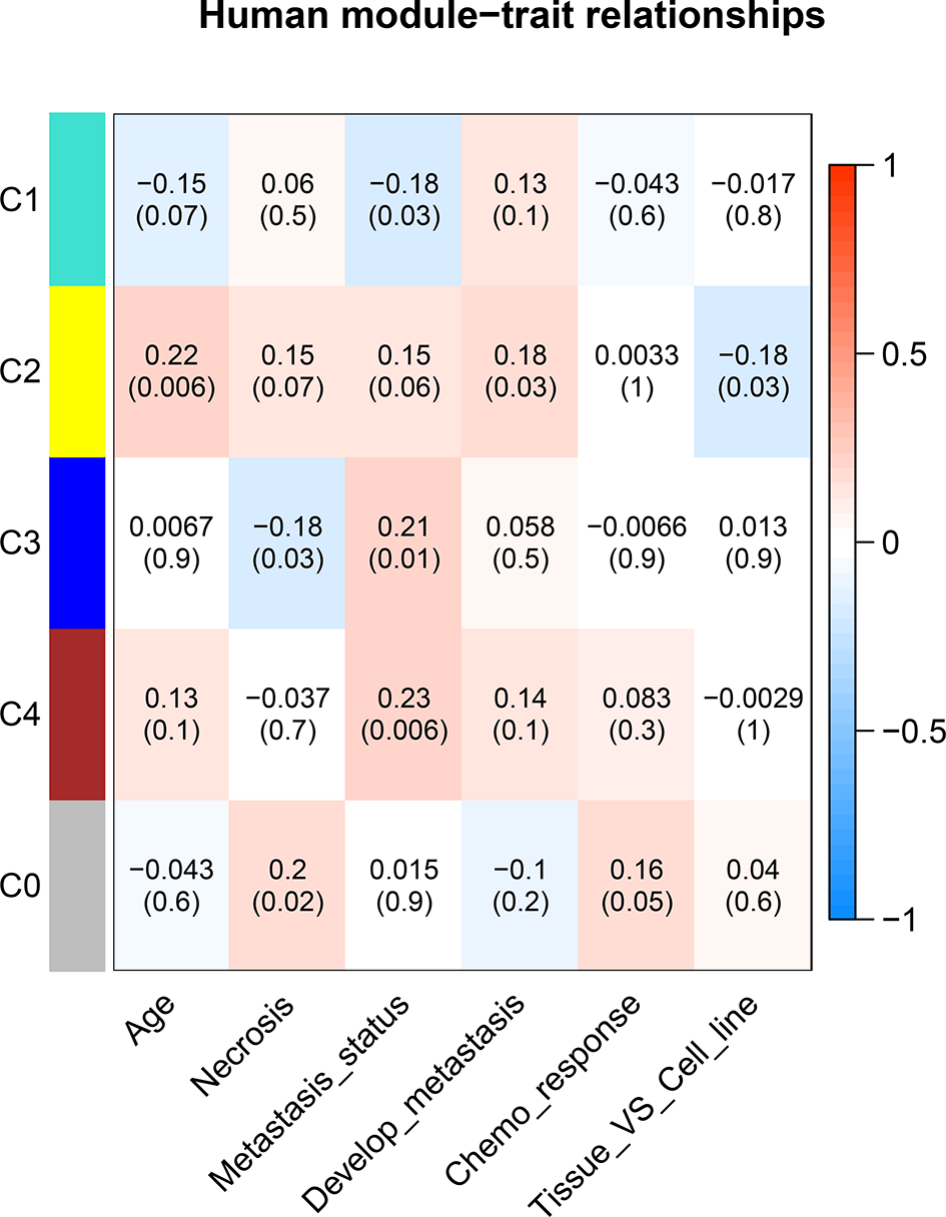
Correlation between consensus modules and clinical traits in humans. The red color represents a positive correlation, the darker the color the higher the correlation. The top number in each cell indicates the correlation coefficient, and the bottom number indicates the correlation significance (*p* value).

To understand the potential function of these modules and their correlation with OS, the correlation between each ME and clinical traits was calculated using Spearman correlation. A certain grade of necrosis resulted in 39 human samples, in which a higher degree was referred to a higher percentage of necrosis. Information regarding primary or metastatic OS tumor was provided in 136 human samples. Twenty-seven samples provided the information whether OS would result in metastasis development in the next 5 years. Forty-eight samples provided the information of chemo response. Whether samples were cell lines or tumor specimens was also correlated with MEs. As regard human clinical traits, C3 was significantly correlated with necrosis. Three modules (C1, C3, and C4) were significantly correlated with tumor status, which was referred to the primary or metastatic tumor, and C2 was significantly correlated with tumor developing metastasis (**Figure 5**).

### Functional Annotation of Interested Modules

**Figure 6:**
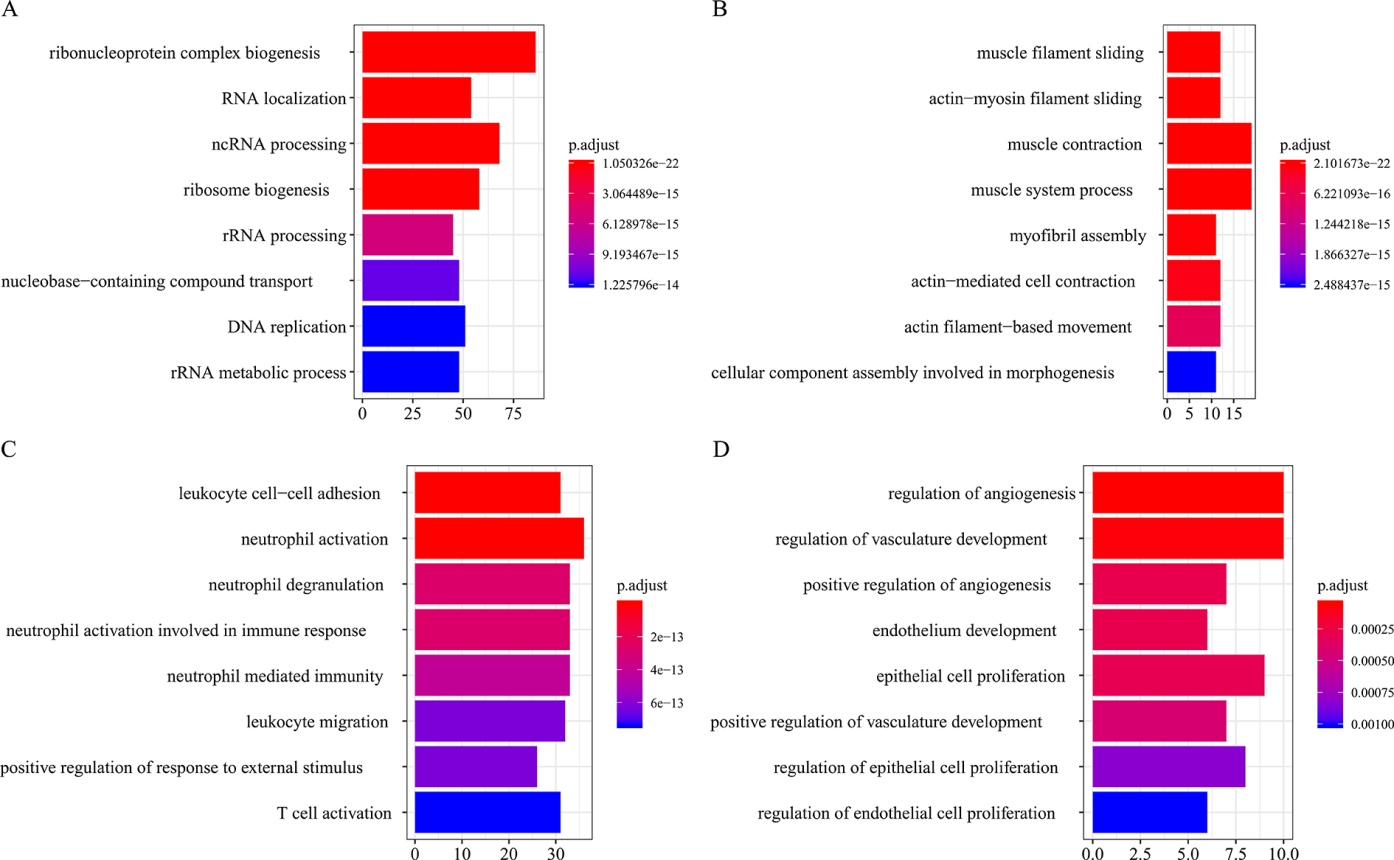
Biological process function annotation of consensus modules (C1–C4: **A**, **B**, **C**, **D**) (enriched cutoff: *p* < 0.01).

**Figure 7:**
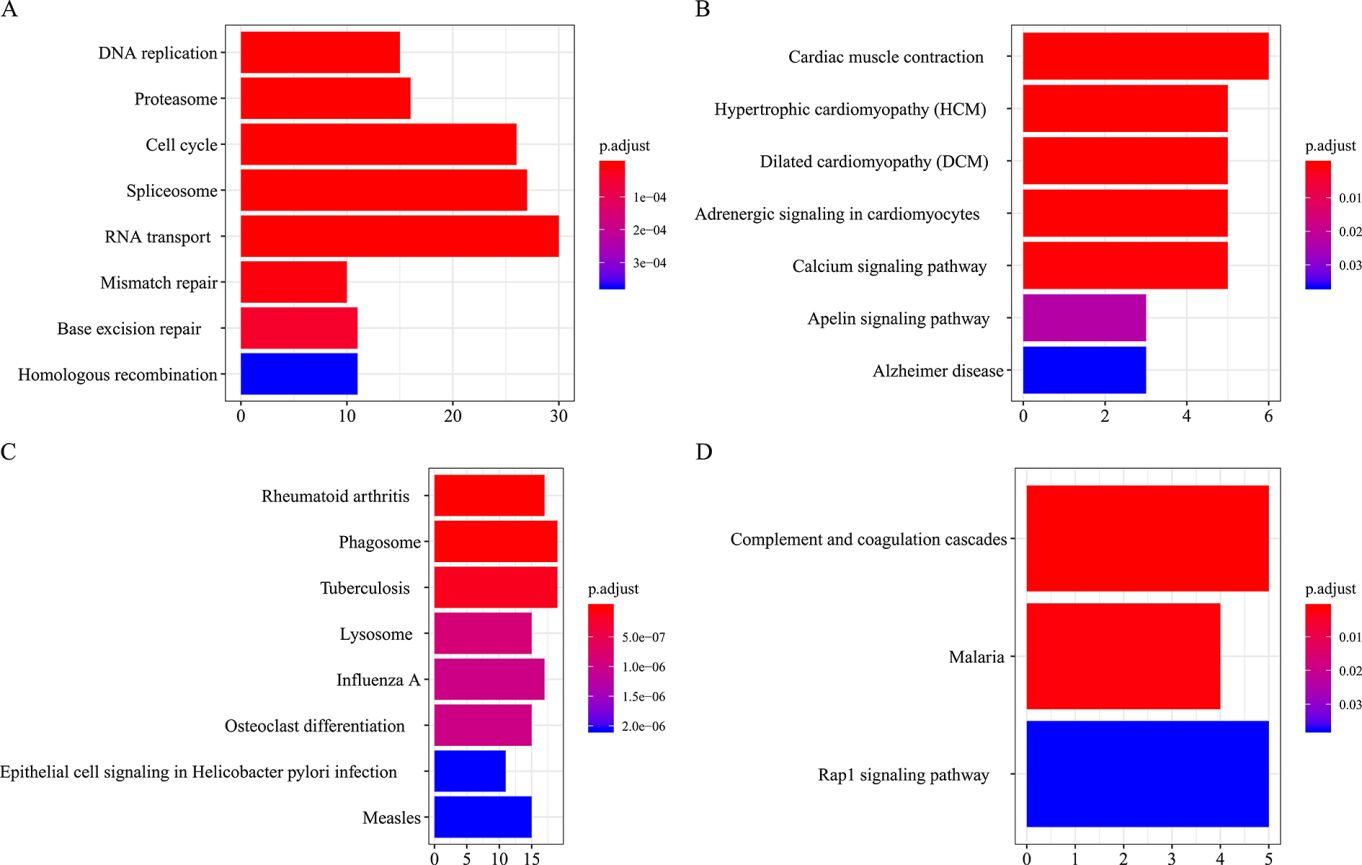
KEGG pathways annotation of consensus modules (C1–C4, **A**, **B**, **C**, **D**) (enriched cutoff: *p* < 0.05).

All the modules in the human network, canine network, and consensus network were annotated to find related KEGG pathways in modules. Detailed annotation information is provided in the **Supplementary Data Sheets 16–30**. The most enriched biological processes and KEGG pathways are shown in **Figures 6** and **7**. The four consensus gene modules showed significantly different biological functions. C1 plays a role in the biosynthesis of macromolecules constituents, assembly, and arrangement of constituent parts of complexes containing RNA and proteins (GO items: ribonucleoprotein complex biogenesis, RNA localization, ncRNA processing and ribosome biogenesis; KEGG item: RNA transport, Spliceosome and Cell cycle). C2 is involved in the muscle system process (GO item: muscle system process and muscle contraction; KEGG item: cardiac muscle contraction). C3 participates in the cellular immune response (GO item: neutrophil activation and leukocyte cell–cell adhesion; KEGG item: phagosome and rheumatoid arthritis). C4 comes into play in angiogenesis (GO item: regulation of angiogenesis and regulation of vasculature development).

### PPI Network and Hub Genes

**Figure 8:**
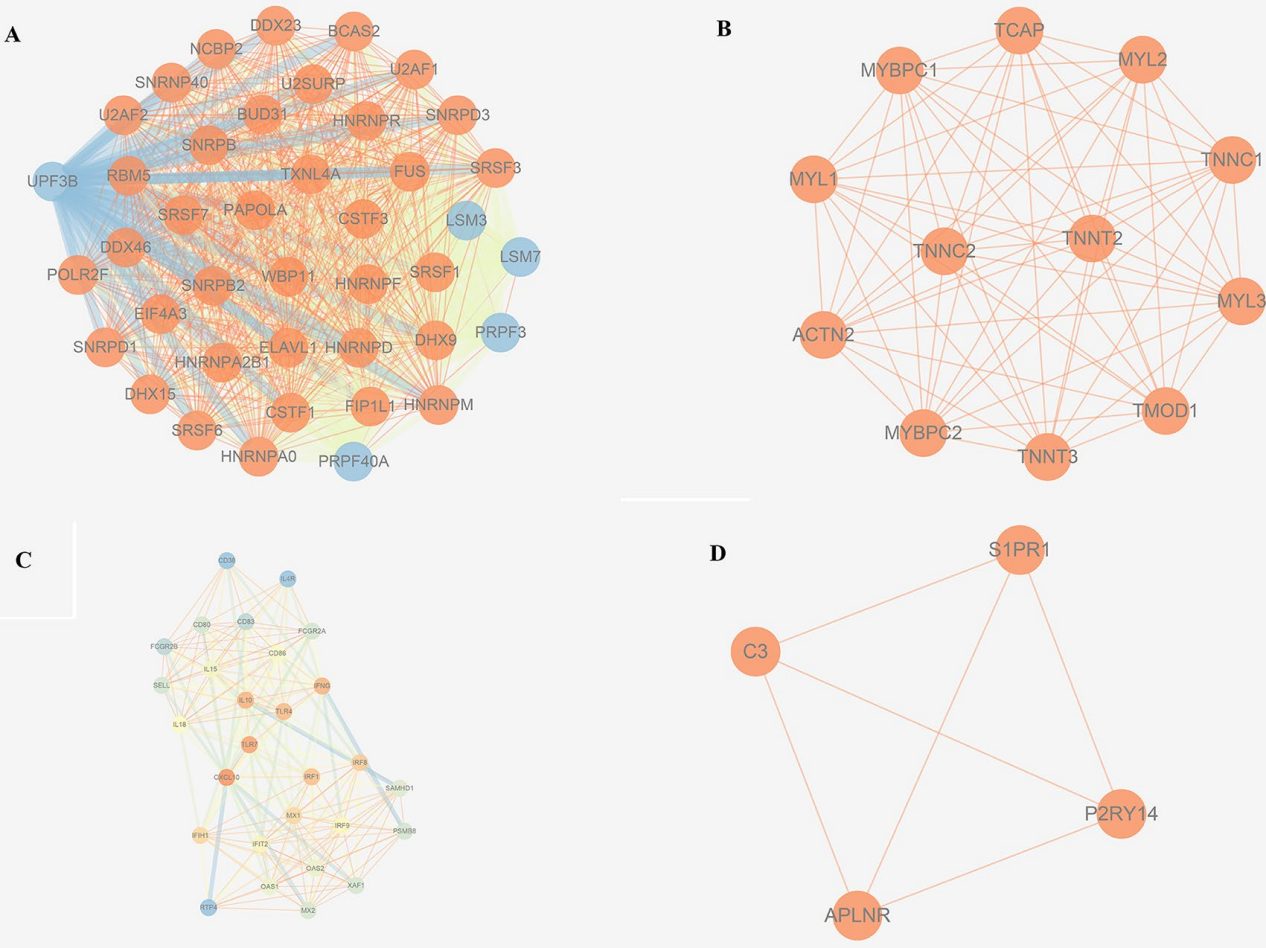
PPI hub networks of each consensus modules. **(A)** C1; **(B)** C2; **(C)** C3; **(D)** C4.

The trait-associated modules were imported into the STRING database, and PPI network was built according to the known protein interactions. PPI hub network was composed of nodes that were closely connected with each other and with the center of the whole module PPI network, and the destruction of these nodes (or hub genes) might have an impact on the whole PPI network, and correlated with most of the genes in the WGCNA gene module. Thus, hub genes could affect the biological function of the whole module of OS. PPI hub networks of each consensus module are shown in **Figure 8**. Hub genes of consensus modules are listed in **Supplementary Sheet 30**. As a result, no hub gene was identified in C1, and a total of 9 hub genes were identified in C2, 18 were identified in C3, and 1 hub gene was identified in C4.

### Hub Modules and Genes Resulted in a Significant Correlation With Survival in Human

**Figure 9:**
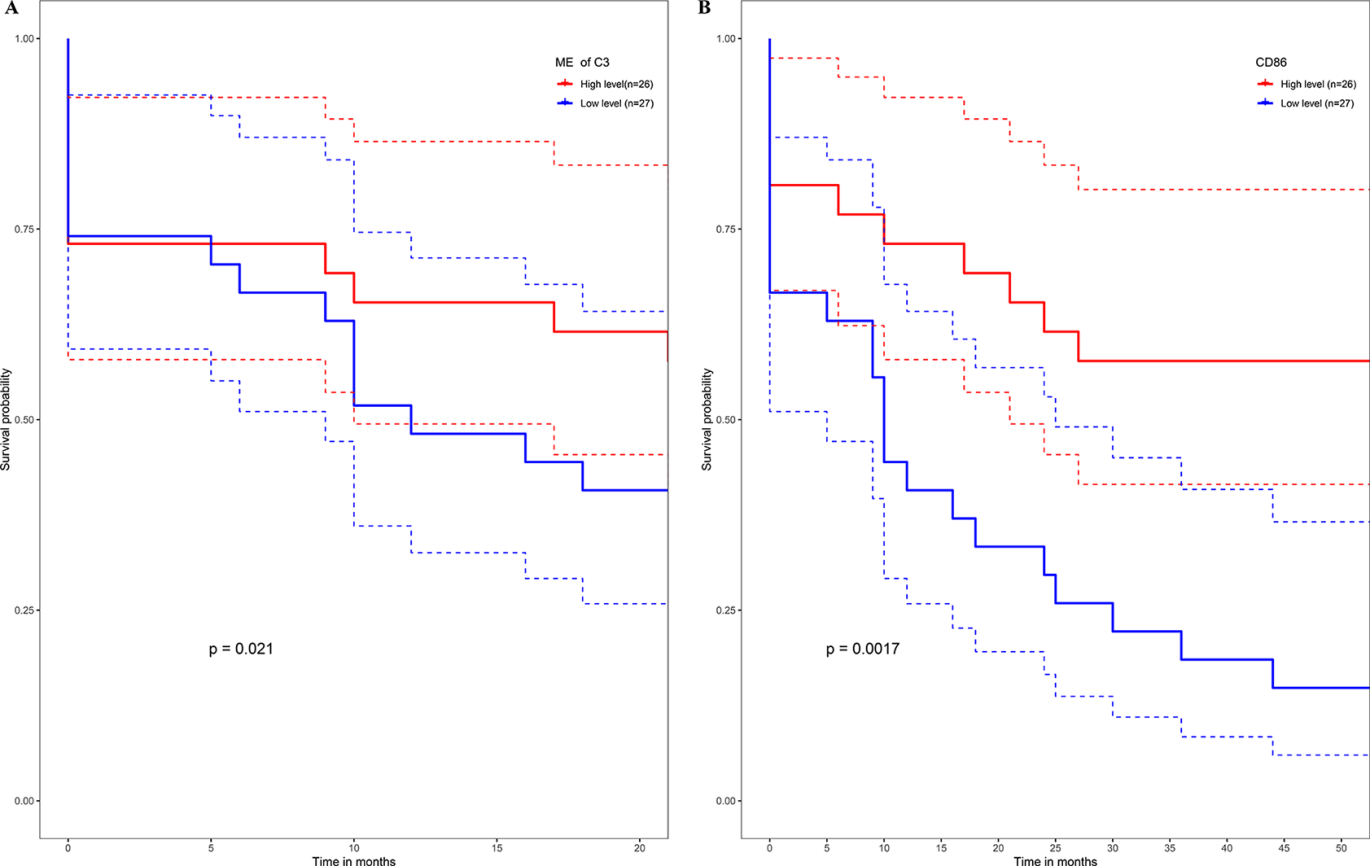
High expression level of C3 ME **(A)** or CD86 ME **(B)** showing significant higher rate of metastasis-free survival. (Red solid line: survival curves of high expression samples of selected gene in comparison with the median expression level. Blue line: survival curves of low expression samples of selected gene in comparison with the median expression level. Dotted line: the upper and lower limits of 95% confidence interval).

No metastasis-associated survival information was provided by the training data set. In consideration of the high conservation in human training data set and test data set (**Supplementary Figure 3**), in the latter data set MEs of consensus modules were recalculated. The recalculation showed that in the test data set, samples with higher level of C3 ME possessed a significant higher rate of metastasis-free survival in comparison to the median level of C3 ME (**Figure 9A**). In our attempt to evaluate if hub genes in modules actually have a role in survival, the results showed that when compared with median lever, higher CD86 expression corresponded to a significantly higher metastasis-free survival rate in human samples (**Figure 9B**).

### Networks Validation in Mouse Species

**Figure 10:**
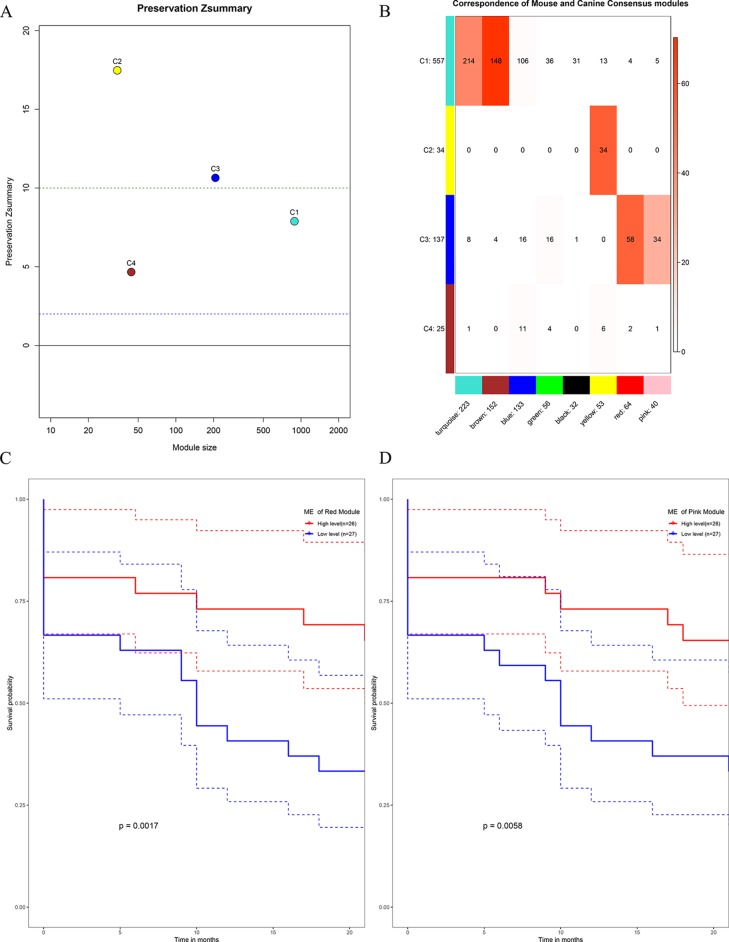
Preservation test of human–canine consensus modules in mouse expression data set **(A)**. The higher the value, the more conservative it was, below cutline 2 indicating no evidence of preservation, upon cutline 10 indicating strong evidence of preservation. Consensus module 3 of human–canine network showed a significant overlap with red and pink consensus modules of human–mouse network **(B)**. High expression level of red ME **(C)** and pink ME **(D)** of human–mouse network showing significant higher rate of metastasis-free survival. (Red solid line: survival curves of high expression samples of selected gene in comparison with the median expression level. Blue line: survival curves of low expression samples of selected gene in comparison with the median expression level. Dotted line: the upper and lower limits of 95% confidence interval).

All the human–canine consensus modules showed moderate to strong evidence of preservation (Preservation Zsummary 4.7-17) in the mouse expression data set (**Figure 10A**). A total of eight co-expression modules were identified in human-mouse consensus network. The original C3 module of human–canine consensus network showed a significant (*p* < 0.05) overlap with the consensus red and pink modules of human–mouse consensus network (**Figure 10B**). Both consensus red and pink modules of human–mouse network showed metastasis-free survival significance (*p* < 0.05) in external data set GSE21257 (**Figures 10C, D**). As for the differential part, 753 of 1,506 module genes of human–canine model were not assigned to any consensus module in human–mouse model. Human–canine model-specific genes of C1 enriched in function of metabolic process. All the genes in C2 were shared by the module genes of human–mouse model. Human–canine model-specific genes of C3 enriched in function of response to external stimulus, and Toll-like and NOD-like receptor signaling pathways. Human–canine model-specific genes of C4 enriched in function of endothelium development, and Rap1, MAPK, and PI3K-Akt pathways. All the human–canine model-specific genes and enrich results were provided in **Supplementary Sheets 31–40**.

## Discussion

OS is one of the most malignant tumors in children and adolescents, with a high tendency of developing metastasis. Treatment choices for metastasis are limited, and the survival rate is poor. Therefore, there is an urgent need to discover the mechanism of OS metastasis and hub nodes in the development of this disease. Due to the complexity of the living environment and gene expression noise, it is usually difficult to discover the “real” reasons behind the disease. Fortunately, animal modules can give us some hints, thanks to the comparable genetic information and simplified living environment. OS naturally occurs in both human beings and canines, showing similarities in the disease process. Thanks to these aspects, a co-expression analysis was conducted to identify preserved gene modules across species function annotation, and correlation analysis was performed to find out potential associations between the preserved modules and clinical characteristics. Subnetwork analysis further revealed the hub nodes in the preserved modules, and then the external data set was used for validation. Finally, C3 and hub gene CD86 were found as having a significant correlation with metastasis-free survival in OS.

As regard single species, gene correlation in all the human modules could be found in the canine species, thanks to the high preservation of human modules in canine species, although two (CA2, CA4) of the eight modules constructed in canine showed low conservation in human species. Thus, these two modules were actually canine-specific modules. A further function annotation revealed the metabolic pathways of these two modules (**Supplementary Sheets 20 and 22**). These evidences suggested the existence of some metabolic differences between OS in dogs and humans during evolution.

As regard the consensus modules, they were constructed according to the similar gene relationship in both species. Modules were shared by both species and showed high preservation across species. Correlation analysis of consensus modules and clinical traits could reveal the relationship between MEs expression level and clinical characteristics. Three consensus modules (C1, C3, C4) were significantly correlated with OS metastatic status, and C2 module was significantly correlated with the development of metastasis (**Figure 5**). To further validate the results, an external data set was used. In the representation of the gene module C3, higher C3 ME expression showed a significant higher rate of metastasis-free survival.

Functional enrichment analysis of gene modules revealed that C1, C2, C3, and C4 were associated with function of RNA synthesis, muscle contraction, cellular immune response (especially neutrophil-mediated immune responses), and angiogenesis, respectively, and they were all associated with metastasis.

Survival information could reflect the overall impact of gene modules on OS. Unfortunately, no metastasis-associated information was provided in the training data sets. Instead, the Illumina platform’s data set GSE21257 as test data set was used, which was the largest data set we found containing metastasis-related survival information of OS. Consensus co-expression gene modules showed well conservative prosperity in the test data set. Clinical analysis revealed that only C3 showed metastasis-free survival significance (*p* = 0.021). Moreover, one of the hub gene, such as CD86, identified by module and PPI network analysis in the training data set, showed metastasis-free survival significance (*p* = 0.0017). Unfortunately, other hub genes (see **Supplementary Sheet 30**) did not affect the metastasis-free survival (data not shown).

To further validate the human–canine consensus network, the expression data of mouse species was used. Module membership of human-canine consensus modules could be reproduced on the expression data of mouse to some extent. Then, we constructed the human–mouse consensus network and compared with the original human–canine consensus network. Human–mouse red and pink modules, which showed a significant overlap with human–canine consensus modules 3 (C3), also showed prognosis significance. These results suggest that the network structure identified in the human–canine model was stable across multiple species. Enrichment results indicate that there were some differences in OS metabolism, response to external stimulus, and endothelium development between the two animal models. Moreover, some cancer-associated pathways, such as Rap1, MAPK, and PI3K-Akt signaling pathways, were identified in human–canine model but not human–mouse model. Above results indicate that canine animal model may be a better OS model, which can mimic more characteristic of the disease.

As mentioned above, C3 showed a significant higher rate of metastasis-free survival in OS; thus, our attention was focused on the function of C3. Neutrophils are the primary immune cells that protect the body from microbial infection and eliminate pathogens. Neutrophils derive from bone marrow hematopoietic stem cells. After differentiation and development in bone marrow, neutrophils enter the blood stream or tissues, accounting for about 50% to 70% of the total number of peripheral blood leukocytes. In recent years, studies showed that neutrophils play a dual role in tumors (Zhang et al., 2016). A recent study found that tumor-associated neutrophils (TANs) constitute the 5% to 25% of cells isolated from the digested human lung tumors. Compared with blood neutrophils, TANs display an activated phenotype. Functionally, both TANs and neutrophils isolated from distant nonmalignant lung tissue are able to stimulate T-cell proliferation and IFN-γ release. Cross-talk between TANs and activated T cells lead to a substantial upregulation of costimulatory molecules on the neutrophil surface, which supports T-cell proliferation in a positive-feedback loop, thus inhibiting tumor cell survival (Eruslanov et al., 2014). The colon microbiota and microbial dysbiosis drive colon tumorigenesis. Another study showed that in mice, neutrophils can reduce the growth and invasion of colon tumors by restricting tumor-associated microbiota (Triner et al., 2019). Our study found that C3 was highly associated with metastasis-free survival in OS; thus, probably neutrophils play an important role in this mechanism.

CD86 is also known as B7.2. Its principal mode of action is by binding to the cluster of differentiation 28 (CD28). Along with the cluster of differentiation 80 (CD80), these molecules provide the necessary stimuli to prime T cells against antigens presented by antigen-presenting cells (Collins et al., 2005). Previous studies showed that cancer cells are potential antigen-presenting cells. CD80 and CD86 are moderately expressed in some tumors, such as non-small cell lung cancer, especially on the surface of cancer cells, thus helping cancer cells to escape from the immune attack. However, CD80 expression is higher in pancreatic carcinoma tissues than in normal pancreatic tissues, and CD80 is significantly correlated with the pathological grade and tumor-node-metastasis stage (Wang et al., 2010). CD80 and CD86 have similar functions; thus, we speculated that CD86 might function as a biomarker for OS metastasis, although the relationship between CD86 and OS needs further study.

This study possesses some limitations. First, sample sizes were relative small in each study and, as a consequence of that, different ways of sample processing might result in technical noises. Second, the clinical information was limited; only a small portion of samples provided disease-associated information, thus weakening the power of the correlation analysis. Third, due to the difference of probes design across species, only nearly 4,000 common genes were identified, with a risk of missing important genes. Therefore, a further, single-center large sample study of standardized processes should be performed to validate the findings in this study, although our study gave new insights regarding this disease.

## Data Availability

Publicly available datasets were analyzed in this study. This data can be found here: http://www.ncbi.nlm.nih.gov/geo.

## Author Contributions

ZZ and DY designed the study. ZJ and SL performed the data collection. PZ, MT, and YW performed the data analysis. ZJ, XZ, and DL drafted the manuscript. All authors read and approved the final version of the manuscript.

## Funding

The work was supported by the National Natural Science Foundation of China (81571530) and the Fundamental Research Funds for the Central Universities.

## Conflict of Interest Statement

The authors declare that the research was conducted in the absence of any commercial or financial relationships that could be construed as a potential conflict of interest.
