# Supplementary material for: Cross-Species Gene Expression Analysis Reveals Gene Modules Implicated in Human Osteosarcoma
Source: Front Genet. 2019 Aug 7;10:697. doi: 10.3389/fgene.2019.00697 (PMC6693360; doi:10.3389/fgene.2019.00697)
Supplement: Supplementary file 1 [file Table_1.docx]

Aggregation of expression data

Collapse rows into single gene maximum mean measurement

Universal Ensemble annotation of probe identifiers

Limma

Affymetrix Human Gene 1.0 ST Array

Affymetrix Human Genome U133 Plus 2.0 Array

RMA background

correction

Cyclic loess normalization

Affymetrix Canine Genome 1.0 Array

Raw expression study files

Affymetrix Canine Genome 2.0 Array

Studies pass qualitative inclusion criteria

Intersect all datasets in Canine or Human on common Ensemble gene identifiers

Canine

Samples:117

Genes:7081

Human

Samples:154

Genes:12730

Batch effect remove and Z-score normalization within specifies

Common genes identified across specifies based on homologous sequence

Merged Canine or Human expression set input for WGCNA

Common gene:4609

Quality assessment of all genes and samples

Extract genes

Test survival significance of MEs in external dataset

Export network structure (TOM) and annotated modules to Cytoscape

Select modules of interest

Correlate eigengenes with traits

Dynamic tree-cutting & module detection

Calculate species-specific module eigengenes and consensus modules

Blockwise network construction using 1-TOM + user defined settings

Create adjacency matrix

Assess scale-free topology:

Choose soft-thresholding power(β)

Outliers identification through a samples cluster process

Hub genes

STRING

Protein-protein interaction

Functional annotation

Extract genes

CYTOSCAPE

Network visualization

Canine or Human expression set
