# Supplementary material for: Cross-Species Gene Expression Analysis Reveals Gene Modules Implicated in Human Osteosarcoma
Source: Front Genet. 2019 Aug 7;10:697. doi: 10.3389/fgene.2019.00697 (PMC6693360; doi:10.3389/fgene.2019.00697)
Supplement: Supplementary file 2 [file Presentation_1.pdf]

# Supplementary Figures

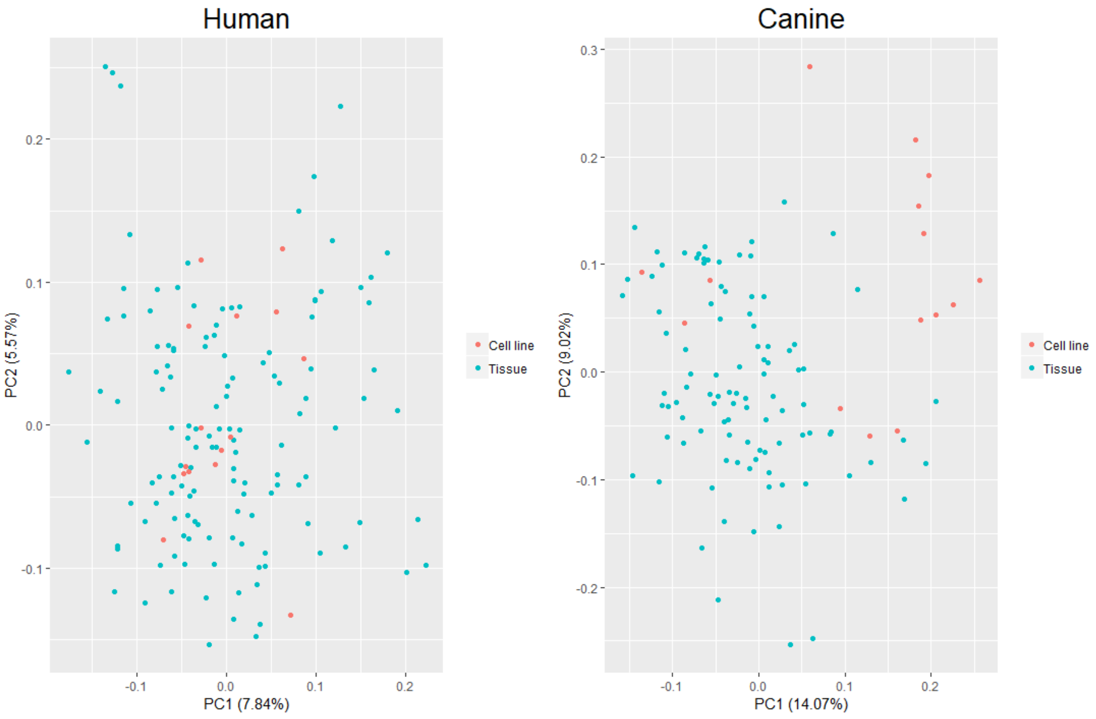


**Supplementary Figure 1:** PCA plot for human and canine expression data.

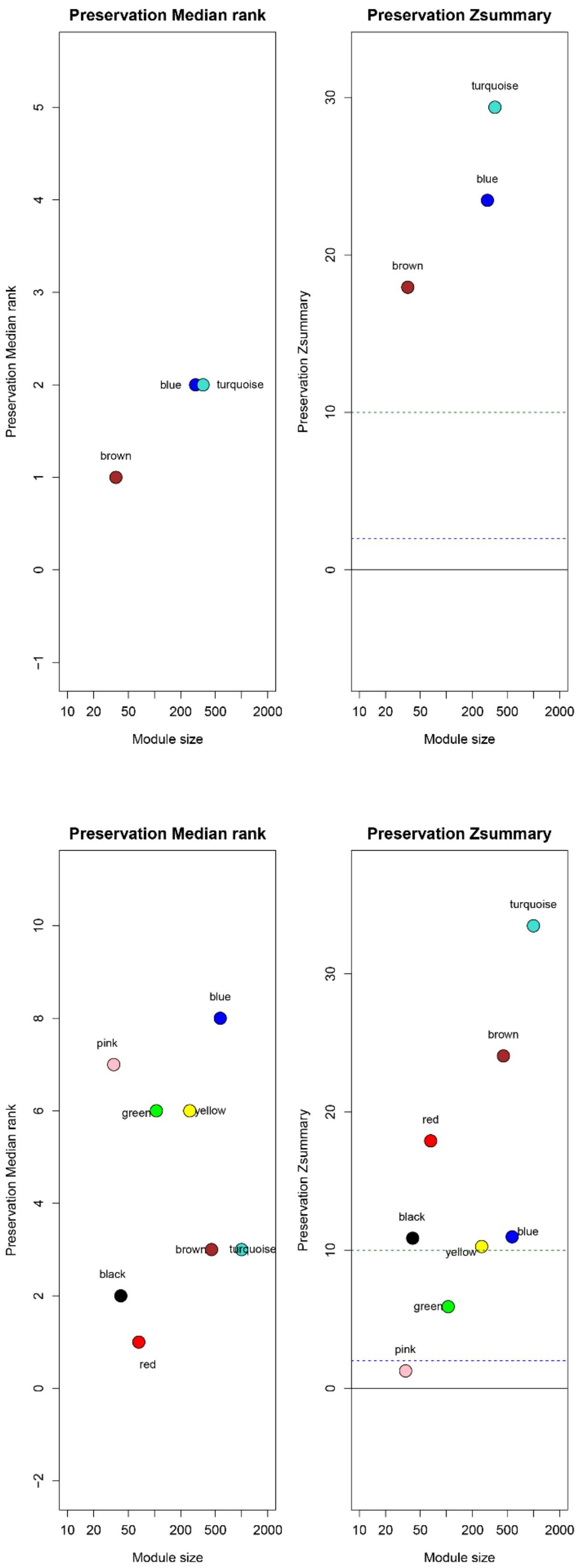


**Supplementary Figure 2:** Preservation test of human modules in canine (top), canine modules in human (bottom).

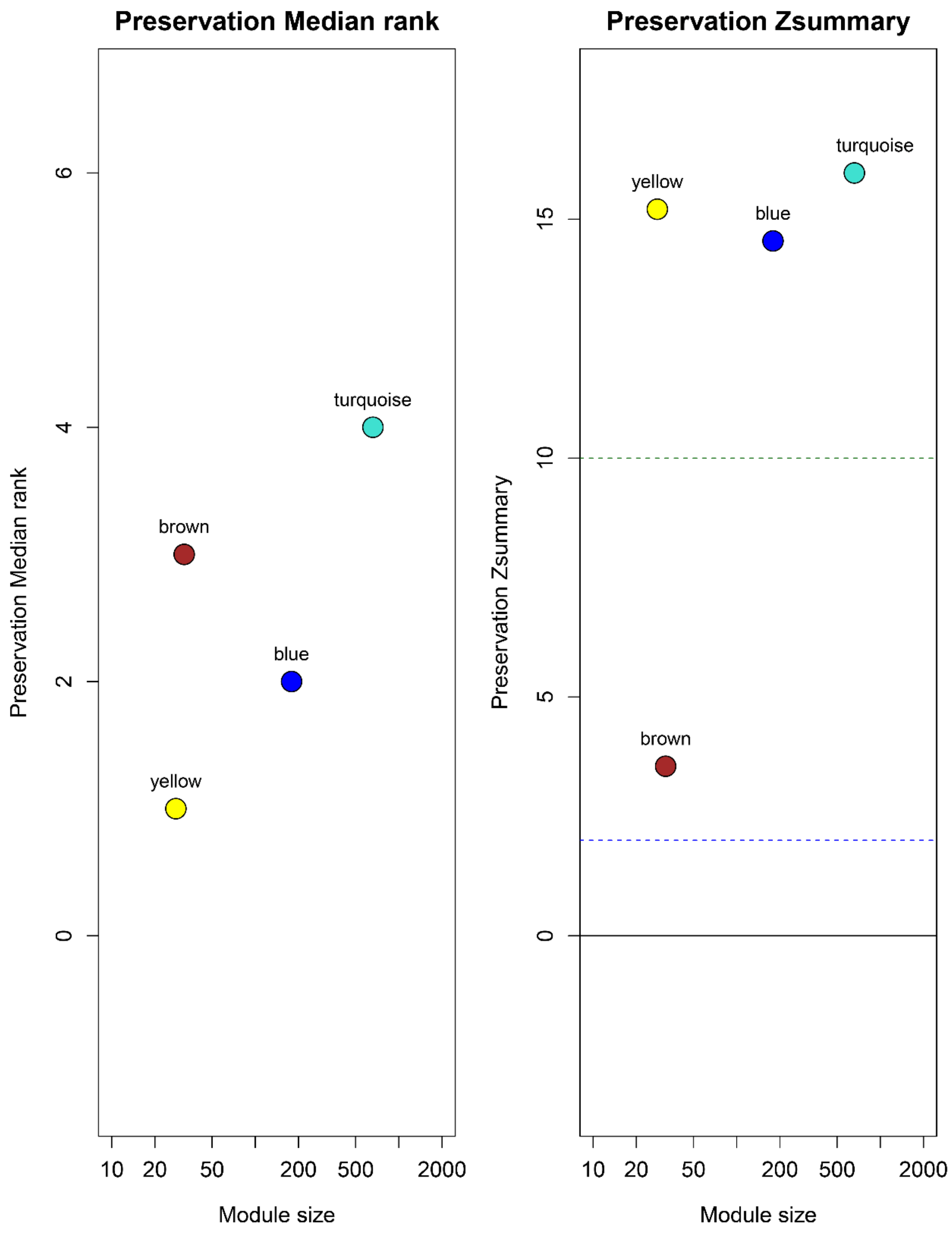


**Supplementary Figure 3:** Preservation test of consensus modules in the test human dataset (Color reference:C1-turquosise, C2-yellow, C3-blue, C4-brown).
